# Can hearing handicap be linked to frailty? A cross-sectional study

**DOI:** 10.1590/2317-1782/e20240107en

**Published:** 2025-02-07

**Authors:** Ruana Danieli da Silva Campos, Henrique Pott, Marisa Silvana Zazzetta, Fabiana de Souza Orlandi, Sofia Cristina Iost Pavarini, Ariene Angelini dos Santos-Orlandi, Isabela Thaís Machado de Jesus, Karina Gramani Say, Aline Cristina Martins Gratão, Letícia Pimenta Costa-Guarisco

**Affiliations:** 1 Pós-graduação em Gerontologia, Universidade Estadual de Campinas – UNICAMP - Campinas (SP), Brasil.; 2 Departamento de Medicina, Universidade Federal de São Carlos – UFSCar - São Carlos (SP), Brasil.; 3 Departamento de Gerontologia, Universidade Federal de São Carlos – UFSCar - São Carlos (SP), Brasil.; 4 Departamento de Enfermagem, Universidade Federal de São Carlos – UFSCar - São Carlos (SP), Brasil.

**Keywords:** Aged, Frailty, Cognition, Social Support, Hearing Loss

## Abstract

**Purpose:**

To investigate potential association between different types of frailty and hearing handicap in the older population.

**Methods:**

A study was conducted on frailty among older adults in the context of social vulnerability. The study involved 229 participants who underwent physical, cognitive, and social frailty assessments. Physical frailty was assessed using Fried's Frailty Phenotype, while cognitive frailty was characterized by the presence of physical frailty and cognitive decline. The Makizako index was used to assess social frailty, and the HHIE-S questionnaire was applied to quantify hearing handicap. Participation restrictions related to hearing difficulties were explored in relation to the three types of frailty using logistic regression.

**Results:**

Hearing handicap were found to be associated with physical, cognitive, and social frailties. However, in a multivariate binary logistic regression analysis, the emotional scale of HHIE-S was only a predictive factor for physical frailty, along with older age, lower education, and the presence of comorbidities. Age and the presence of comorbidities were the only associated explanatory variables for cognitive frailty. Social frailty was only associated with the presence of cognitive changes.

**Conclusion:**

Hearing loss-related participation restrictions can be a significant challenge for older adults. Those who also have an emotional impairment, caused by hearing loss, are even more vulnerable to becoming frail or pre-frail. It’s important to prioritize the needs of this population and provide the necessary support to enhance their quality of life and prevent further decline.

## INTRODUCTION

Age-related hearing loss is one of the most common chronic conditions in older people. It is a result of the natural aging process and is progressive and irreversible, making it the primary cause of years lived with a disability^(1)^. Although it begins around the age of 55, it is not noticeable at first and can result in difficulty understanding speech, leading to limitations in social participation and daily activities^(2)^. Therefore, the World Health Organization (WHO) emphasizes the importance of describing the nature and severity of functional limitations and disability related to hearing conditions, including limitations in activities and social participation, in accordance with the International Classification of Functioning, Disability and Health (ICF)^(3)^.

The term "participation restriction" refers to the social, situational, and emotional effects that are caused by hearing loss. These effects arise due to the recognition of the impact of the hearing handicap, limitation or impediment in carrying out daily activities. People with hearing loss also face changes in their social and functional routines, leading to issues like social isolation and communication difficulties. It is crucial to determine the impact of participation restriction on healthy aging and its association with negative aging outcomes, such as frailty.

Frailty is a major concern among older adults, having negative impacts on their health and the well-being of their families, communities, and healthcare systems. The clinical conditions leading to frailty are well-documented in the literature. Despite numerous studies on the causes, outcomes, and conditions associated with frailty since the Fried et al. proposal of the frailty phenotype in 2001, it wasn't until 2014 that the first study established a link between self-reported hearing handicap and frailty^(4,5)^. While the biomedical perspective has dominated the frailty literature, recent definitions recognize the impo rtance of social conditions. However, social factors of frailty remain underexplored. Social frailty, a term coined in 2017, refers to the risk of losing social and behavioral resources, social activities, and self-management skills, which are necessary to fulfill basic social needs. When such resources are inadequate, individuals are at risk of becoming socially frail^(6)^.

According to several studies, there is a correlation between hearing loss and frailty. In 2022, in a study carried out with the same sample as the present article, authors found that hearing handicap is higher in pre-frail and frail older individuals and greater restriction is associated with higher physical frailty scores^(7)^. Hearing loss is also linked to lower attendance at social gatherings and a feeling of uselessness, leading to greater social isolation^(8)^. Authors reported that hearing loss and social frailty may be associated with mild cognitive impairment in older individuals, possibly due to communication difficulties reducing the ability to engage in cognitively stimulating activities, resulting in social isolation and loneliness^(9)^.

This study aims to explore the impact of hearing handicap on older individuals living in a community with low income and education levels. The research focuses on understanding the restrictions on participation in the biopsychosocial aspects of frailty caused by hearing loss. As frailty plays a crucial role in the aging process, the study investigates the association between different types of frailty and hearing handicap in the older population.

## METHODS

### Study design

This study presents a secondary analysis of data collected during the project titled "Tool for monitoring frailty levels in older people receiving care in primary health care: evaluation of its effectiveness and efficiency". The Ethics Research Committee of the Federal University of São Carlos (CAAE: 86967418.4.0000.5504) approved the study with decision number 3.101.282, in compliance with the ethical guidelines established by Resolution 510/2016 and regulated by the National Health Council.

### Data collect

The study conducted by the research team involved older adults who were registered in community family health units (USF) and met certain eligibility criteria. The team presented the study to USF and invited eligible participants to join it by signing the Consent Form during home visits. The researchers then conducted data collection in the participants' residence in the period of 2017 and 2018. To be eligible for the study, participants had to be 60 years old or older, registered in a USF served by the Family Health Support Center (NASF), and able to understand and communicate verbally. Individuals with severe cognitive or motor deficits, those who used a wheelchair, or those with terminal illnesses were excluded from the study due to their inability to perform the necessary tests. For more information on the data collection process, refer to Jesus^(10)^.

### Measures and variables

To collect sociodemographic and health information, the researchers created a self-reported questionnaire. The questionnaire contained inquiries about gender (male or female), age (in years), self-declared race, marital status (with or without a partner), education (in years), and comorbidities (such as high blood pressure, diabetes mellitus, cancer, osteoporosis, and stroke). Other evaluations conducted involved assessments related to frailty, cognitive function, and hearing ability.

### Cognitive performance

The cognitive function of the participants was evaluated using the Mini-Mental State Examination (MMSE)^(11)^, which incorporated different cut-off scores based on the years of education. For illiterate older subjects, the cut-off score was 17 points, while for those with 1 to 4 years of education, it was 22 points. For those with 5 to 8 years of education, the cut-off score was 24 points, and for those with more than 9 years of education, it was 26 points. Participants who scored below the cut-off score were classified as having cognitive impairment^(12)^.

### Assessment of hearing and its impact on participation restrictions.

The study utilized the Hearing Handicap Inventory for the Elderly - Screening (HHIE-S) questionnaire, which was originally developed by Ventry and Weinstein in 1983 to assess hearing handicap and its impact on the daily activities of older individuals^(13)^. The questionnaire was translated and validated for use in Brazilian Portuguese^(14)^. It consists of ten questions, each with three answer options: yes (4 points), sometimes (2 points), and no (0 points). The total score is calculated by adding up the scores, and it reflects the emotional and social effects of hearing loss. A higher score indicates a greater hearing handicap or restriction in participating in daily activities.

### Frailty assessments

#### Physical frailty

The Fried frailty phenotype was used to assess physical frailty (PF)^(4)^. This approach evaluates frailty based on five components: unintentional weight loss, fatigue, low handgrip strength, low caloric expenditure, and slow gait. The subject's score on these five criteria determines their classification as not frail, pre-frail, or frail. Those with no score in any of the five criteria are considered not frail, while those with a score in one or two criteria are pre-frail. Subjects with a score in three or more criteria are classified as frail.

#### Cognitive frailty

Cognitive frailty refers to a condition where older individuals exhibit both physical frailty and cognitive decline^(15)^. This condition was identified by the physical frailty phenotype and the MMSE score, and those classified as physically frail with cognitive impairment are categorized as cognitively fragile.

#### Social frailty

To determine social frailty, we utilized the Makizako index^(16)^. This index consists of five questions that pertain to social domains, such as daily social activity, social role, social support, and social relationships. Based on the scores obtained from these questions, we classify older individuals as not socially frail if they scored zero in all five criteria, pre-social frail if they scored in one or two criteria, and social frail if they scored in three or more criteria.

### Statistical analysis

Mean and standard deviation or median and interquartile range were used for continuous data, while absolute and relative frequencies were used for categorical data. The Mann-Whitney and Pearson chi-square tests were used for group comparisons of continuous and categorical variables, respectively.

Logistic regression models were used to assess physical, cognitive, and social frailty as a function of all variables, including evaluating the global score of the HHIE-S questionnaire and its sub-scores on social and emotional scales. Sociodemographic and health characteristics such as age, gender, year of birth, year of education, cognition, and presence of two or more comorbidities were included in the analysis. The stepwise strategy of ambidirectional progression was used to select the most suitable model, with the lowest Akaike Information Criterion (AIC) as the criteria for variable selection. The modeling results were presented as Odds Ratios with corresponding 95% confidence intervals. The Statistical Package for the Social Sciences (SPSS) program (version 21.0) was used for all analyses.

## RESULTS

Out of the 238 older adults initially sampled, only 229 were included in the study as 9 had missing values in one or more variables. Most of the participants were female (58.1%) with a mean age of 72 years, living with partners (60.7%), and having no known comorbidity (55.9%). Most participants had normal cognition (60.7%), and no hearing handicap (72.9%). Moreover, most were considered at least pre-frail in their physical and social frailty assessments (76.4% and 80.3%, respectively). (Table 1)

**Table 1:** Descriptive sociodemographic, health, cognition, hearing handicap and frailty variables, (n=229)

**VARIABLE**	**CATEGORY**	**OVERALL** **(%)**
Sex	Male	96 (41.9)
	Female	133 (58.1)
Age, years		72.0 ± 7.3
Education, years		2.6 ± 2.8
Race	White	115 (49.8)
	Non-white	114 (50.2)
Marital Status	With partner	138 (60.7)
	Without partner	91 (39.3)
Comorbidities	With comorbidities	101 (44.1)
	No comorbidities	128 (55.9)
MMSE*	Altered	90 (39.3)
	Normal	139 (60.7)
HHIE-S**	With hearing handicap	62 (27.1)
	Without hearing handicap	167 (72.9)
*Frailty assessments*		
Physical frailty	Pre-frail and Frail	175 (76.4)
	Non-frail	54 (23.6)
Cognitive frailty	Pre-frail and Frail	73 (31.9)
	Non-frail	156 (68.1)
Social frailty	Pre-frail and Frail	184 (80.3)
	Non-frail	45 (19.7)

* Mini-Mental State Examination;

** Hearing Handicap Inventory for the Elderly - Screening version

### Physical frailty

In the univariate analysis, age, years of education, comorbidities, and emotional and social scales of the HHIE-S were significantly associated with physical frailty. In the multivariate logistic regression model, as shown in Table 2, these factors retained their significance.

**Table 2:** A logistic regression model of physical frailty using selected variables based on best-model fitness

**VARIABLE**	**ODDS RATIO**	**P-VALUE**
	**(95% CONFIDENCE INTERVAL)**	
Age, years	1.05 (1.00 – 1.11)	0.03
Education, years	0.82 (0.72 – 0.93)	0.03
Presence of comorbidities	2.85 (1.40 – 5.81)	<0.001
Score on the HHIE-S Emotional Subscale	1.10 (1.00 – 1.22)	0.05

### Cognitive frailty

Age, the presence of comorbidities, and emotional and social scales of HHIE-S were significantly associated with cognitive frailty in a preliminary analysis. However, in a more detailed multivariate logistic regression analysis, only age and the presence of comorbidities remained significant predictors of cognitive frailty, as shown in Table 3.

**Table 3:** A logistic regression model of cognitive frailty using selected variables based on best-model fitness

**VARIABLE**	**ODDS RATIO**	**P-VALUE**
	**(95% CONFIDENCE INTERVAL)**	
Age, years	1.10 (1.05 – 1.15)	<0.001
Presence of comorbidities	2.69 (1.44 – 5.00)	<0.001

### Social frailty

Age, marital status, comorbidities, and emotional and social scales of HHIE-S were initially linked to social frailty. However, a more detailed analysis showed that only an altered MMSE test result remained significantly associated with social frailty. The odds ratio was 0.89 with a 95% confidence interval of 0.80 to 0.97 and a p-value of 0.01.

## DISCUSSION

The study aimed to explore the association between various types of frailties (physical, cognitive, and social) and the hearing handicap. The results revealed that pre-frail and frail individuals tend to have a higher level of restriction on social and emotional scales than non-frail individuals. However, the multivariate logistic regression model showed that different variables were associated with each type of frailty.

Individuals with physical frailty may experience hearing handicap in emotional scale, as indicated by our findings. We observed that for each additional point on the emotional scale, the likelihood of an individual belonging to this group increased by 1.10 times (p=0.05). This association may be attributed to factors such as social isolation, cognitive load, stress, or reduced awareness of the hearing environment^(5,17)^. It is noteworthy that the multivariate model utilized in this study also considered age, education, and comorbidities as important variables.

The relationship between hearing loss and frailty processes has been well-established by current literature, with studies showing that hearing impairment in pre-frail older people can lead to greater risk of frailty progression, regardless of other factors such as gender, age, income, education, cardiovascular diseases, cognition, depression, and socialization^(5,17-19)^. Age has also been identified as a risk factor for frailty progression, regardless of the severity of hearing loss^(20)^. In addition, recent research has shown that hearing impairment in the older, particularly when associated with comorbidities and poor quality of life, can accelerate the progression of frailty^(21)^.

Our results showing the connection between hearing handicap and physical frailty align with recent literature. They emphasize the significance of paying attention to hearing loss and its functional consequences. Hearing impairments can be linked to physical frailty, as both share neuropathological etiologies, as found in the initial studies that pointed out the relationship between hearing and frailty^(5,17)^.

However, in contrast to the hypothesis of the current study, the hearing handicap was not found to be a factor associated with cognitive and social frailties in the logistic regression models, even though it differed between groups with and without frailties.

A study conducted with older individuals demonstrated that those with cognitive frailty have a greater risk of hearing loss compared to those who are healthy or only have physical frailty. The authors suggest that this is due to the common underlying causes of hearing loss and cognitive fragility, such as microvascular diseases, inflammation, and metabolic dysfunction^(20)^. Previous studies have also supported this shared neuropathological etiology hypothesis, providing further evidence and potential explanations for the association between hearing loss and cognitive frailty^(5,15,17,18)^. Authors propose that the shared cause is related to aging, which leads to the deterioration of cognitive and non-cognitive processes, indicating that the declines are shared among multiple and different functions. Therefore, both conditions are the result of a common neurodegenerative process in the aging brain, although there is no direct causality^(22)^.

There are three hypotheses regarding the possible relationship between hearing loss and cognitive decline^(23)^. The first hypothesis suggests that hearing loss and cognitive decline are not necessarily linked. The second hypothesis, known as "cascade," proposes that hearing deprivation directly affects cognition due to low sensory input, and the negative outcomes of hearing restriction, such as decreased socialization, social isolation, loneliness, low verbal communication, and depression. The third hypothesis, "cognitive load," suggests that low hearing contributes to low brain function, which overloads the brain's structure, causing further neurodegeneration. Consequently, hearing loss causes a deficiency of cognitive reserve, leading to impaired cognition^(23)^. Hearing loss is one of the potential risk factors that can lead to dementia, but hearing rehabilitation, such as the use of a hearing aids, can serve as a protective factor^(24)^. Authors suggest that auditory rehabilitation can help prevent cognitive decline^(23-25)^.

Hearing impairment can not only lead to communicative limitations, such as social and family isolation, restrictions on hearing participation and activities of daily living. But it also causes a reduction in the levels of older quality of life. Thus, it was proposed to evaluate the association of social fragility and participation restrictions related to hearing loss.

Although our study found an association between social frailty and hearing handicap in the univariate analysis, the multivariate logistic regression revealed that only cognitive decline was significantly associated with social frailty. However, some findings differed from ours. They discovered that older individuals with social impairment were more likely to experience hearing complications, even after controlling for confounding variables such as age, place of residence, economic status, smoking, and depressive symptoms^(8)^. Their results revealed that older women with hearing loss had a 2.13 times greater risk of being socially frail. According to the authors, low social participation and communication are closely linked to negative outcomes in both social frailty and restricted hearing participation. It is crucial to note that the population examined by the authors was not socioeconomically vulnerable, which may explain the difference in results between the two studies^(8)^.

Moreover, in 2022, authors found that self-reported hearing limitation was greater in pre-frail and socially frail people (OR = 1.78; 95% CI: 1.04-3.06)^(26)^. However, a gender interaction was detected with an association only for the female gender. A possible explanation was that women tend to give more importance to communication and socialization than men. Another explanation, according to the authors, may be the use of self-reported measures, as used in the present study, suggesting that women are more aware of their functional limitations. It is crucial to highlight that our sample consisted of older adults from a socially vulnerable region, facing exclusion, discrimination, and reduced participation in social groups. As a result, we can consider them a group already experiencing social fragility, which could explain why social fragility did not emerge as a significant factor in relation to restrictions on participation. To better understand this association, it is essential to replicate and expand the study to other social groups with different sociodemographic characteristics.

As this was a secondary study that used data from a larger project with diverse objectives and various assessment instruments, individuals with communication difficulties were excluded from the sample. This may have limited the inclusion of those with more severe cognitive deficits, pronounced hearing loss, or extreme physical conditions. Despite higher HHIE-S values in the frail/pre-frail groups compared to the non-frail groups, the slight hearing handicap is noteworthy, as even mild hearing impairment can have negative outcomes such as physical frailty. Hence, it is crucial to investigate the relationship between hearing loss and negative consequences in the aging process, not only in cases of significant hearing handicap but also in mild to moderate levels. The study reinforces the significance of identifying hearing loss early and monitoring hearing handicap to prevent such outcomes. Health professionals who care for older adults should investigate and monitor hearing complaints and their symptoms. The literature recommends early use of sound amplification devices as the best way to prevent hearing handicap and other outcomes associated with hearing loss related to aging, such as frailties and cognitive loss^(23,24,27,28)^.

While knowing the audiometric profile of a sample is crucial in hearing studies, it doesn't provide insight into the effects of hearing loss on the social participation and lives of older adults. This is due to various factors such as the social environment, cognitive and functional reserve, and adaptation capacity of an individual. Instead of audiometry, the HHIE-S instrument was used to gauge the social and emotional losses caused by hearing loss, providing a more functional perspective of the hearing conditions of the sample. It's important to recognize the advancements in knowledge related to frailty and the significance of identifying them to prevent negative outcomes of aging, not just physically but also cognitively and socially.

Older adults who face hearing handicap, especially those with emotional impairments, are more prone to being frail or pre-frail compared to those without such limitations, as per our research. For this population, hearing handicap can be a major obstacle, and it is crucial to focus on their needs and provide adequate support to enhance their quality of life and prevent further deterioration.

## References

[1] Haile LM, Kamenov K, Briant PS, Orji AU, Steinmetz JD, Abdoli A (2021). Hearing loss prevalence and years lived with disability, 1990-2019: findings from the Global Burden of Disease Study 2019. Lancet.

[2] Camargo C, Lacerda ABM, Sampaio J, Lüders D, Massi G, Marques JM (2018). Percepção de idosos sobre a restrição da participação relacionada à perda auditiva. Distúrb Comun.

[3] OMS: Organização Mundial da Saúde (2003). CIF: Classificação internacional de funcionalidade, incapacidade e saúde.

[4] Fried LP, Tangen CM, Walston J, Newman AB, Hirsch C, Gottdiener J (2001). Frailty in older adults: evidence for a phenotype. J Gerontol A Biol Sci Med Sci.

[5] Kamil RJ, Li L, Lin FR (2014). Association of hearing impairment and frailty in older adults. Otolaryngol Head Neck Surg.

[6] Bunt S, Steverink N, Olthof J, Van Der Schans CP, Hobbelen JSM (2017). Social frailty in older adults: a scoping review. Eur J Ageing.

[7] Campos RDDS, Zazzetta MS, Orlandi FDS, Pavarini SCI, Cominetti MR, Santos-Orlandi AAD (2022). Hearing handicap and frailty in community-dwelling older adults living. CoDAS.

[8] Yoo M, Kim S, Kim BS, Yoo J, Lee S, Jang HC (2019). Moderate hearing loss is related with social frailty in a community-dwelling older adults: The Korean Frailty and Aging Cohort Study (KFACS). Arch Gerontol Geriatr.

[9] Bae S, Lee S, Lee S, Jung S, Makino K, Park H (2018). The role of social frailty in explaining the association between hearing problems and mild cognitive impairment in older adults. Arch Gerontol Geriatr.

[10] Jesus ITMD (2021). Frailty of elderly people and associated factors in a vulnerable context: a longitudinal study in primary health care.

[11] Folstein MF, Folstein SE, Mchugh PR (1975). Mini-Mental State: a practical method for grading the cognitive state of patients for the clinician. J Psychiatr Res.

[12] Bertolucci PHF, Brucki SMD, Campacci SR, Juliano Y (1994). O miniexame do estado mental em uma população geral. Impacto da escolaridade. Arq Neuropsiquiatr.

[13] Ventry IM, Weinstein BE (1983). Identification of elderly people with hearing problems. ASHA.

[14] Wieselberg MB (1997). A auto-avaliação do handicap em idosos portadores de deficiência auditiva: o uso do H.H.I.E..

[15] Kelaiditi E, Cesari M, Canevelli M, van Kan GA, Ousset PJ, Gillette-Guyonnet S (2013). Cognitive frailty: rational and definition from an (IANA/IAGG) international consensus group. J Nutr Health Aging.

[16] Makizako H, Shimada H, Tsutsumimoto K, Lee S, Doi T, Nakakubo S (2015). Social frailty in community-dwelling older adults as a risk factor for disability. J Am Med Dir Assoc.

[17] Kamil RJ, Betz J, Powers BB, Pratt S, Kritchevsky S, Ayonayon HN (2016). Association of hearing impairment with incident frailty and falls in older adults. J Aging Health.

[18] Liljas AE, Carvalho LA, Papachristou E, Oliveira CD, Wannamethee SG, Ramsay SE (2017). Self-reported hearing impairment and incident frailty in English community-dwelling older adults: a 4-year follow-up study. J Am Geriatr Soc.

[19] Liu Y, Qian P, Guo S, Liu S, Wang D, Yang L (2022). Frailty and hearing loss: from association to causation. Front Aging Neurosci.

[20] Ruan Q, Chen J, Zhang R, Zhang W, Ruan J, Zhang M (2021). Heterogeneous influence of frailty phenotypes in age-related hearing loss and tinnitus in Chinese older adults: an explorative study. Front Psychol.

[21] Lorenzo-López L, López-López R, Maseda A, Buján A, Rodríguez-Villamil JL, Millán-Calenti JC (2019). Changes in frailty status in a community-dwelling cohort of older adults: the VERISAÚDE study. Maturitas.

[22] Uchida Y, Sugiura S, Nishita Y, Saji N, Sone M, Ueda H (2019). Age-related hearing loss and cognitive decline: the potential mechanisms linking the two. Auris Nasus Larynx.

[23] Stahl SM (2017). Does treating hearing loss prevent or slow the progress of dementia? Hearing is not all in the ears, but who’s listening?. CNS Spectr.

[24] Livingston G, Huntley J, Sommerlad A, Ames D, Ballard C, Banerjee S (2020). Dementia prevention, intervention, and care: 2020 report of the Lancet Commission. Lancet.

[25] Luz VBD, Ghiringhelli R, Iório MCM (2018). Restrições de participação e estado mental: estudo em novos usuários de próteses auditivas. Audiol Commun Res.

[26] Díaz-Alonso J, Bueno Pérez A, Toraño Ladero L, Caballero FF, López García E, Rodríguez Artalejo F (2021). Limitación auditiva y fragilidad social en hombres y mujeres mayores. Gac Sanit.

[27] Mantello EB, Marino MV, Alves AC, Hyppolito MA, Reis ACMB, Isaac MDI. (2016). Avaliação da restrição de participação em atividades de vida diária de idosos usuários de aparelhos de amplificação sonora individual. Medicina (Ribeirão Preto. Online).

[28] Cominetti MR, Pott H, Zúñiga RG, Romero-Ortuno R (2023). Protecting cognitive function in older adults with age-related hearing loss: Insights from The Irish Longitudinal Study on Ageing (TILDA) and the role of hearing aids. Arch Gerontol Geriatr.

